# Incidence of COVID-19 in patients with rheumatic disease: is prior health education more important than shielding advice during the pandemic?

**DOI:** 10.1007/s10067-020-05494-6

**Published:** 2020-11-11

**Authors:** Sarah Kipps, Anindita Paul, Sreekanth Vasireddy

**Affiliations:** Department of Rheumatology, Bolton One Health Centre, Bolton NHS FT, Moor Lane, Bolton, BL3 5BN UK

**Keywords:** COVID-19, Health education, Rheumatic disease, Risk stratification, SARS-CoV-2, Shielding

## Abstract

The COVID-19 pandemic has led to major changes in clinical practice on a global scale in order to protect patients. This includes the identification of vulnerable patients who should “shield” in order to reduce the likelihood of contracting SARS-CoV2. We used national specialty guidance and an adapted screening tool to risk stratify patients identified from our prescribing and monitoring databases, and identify those needing to shield (score ≥ 3) using information from departmental letters, online general practice records and recent laboratory investigations. We collated underlying rheumatological conditions and risk factors. Two months into the shielding process, we examined the COVID-19 status of these patients using hospital laboratory records and compared to population level data. Of 887 patients assessed, 248 (28%) scored ≥ 3 and were sent a standard shielding letter. The most common risk factor in the shielding letter group was age ≥ 70 years and/or presence of a listed co-morbidity (199 patients). The most common rheumatology conditions were rheumatoid arthritis (69.4%), polymyalgia rheumatica (8.5%) and giant cell arteritis (8.5%). Coronavirus incidence rates were similar in the shielding letter group (0.403%) and in the UK population (0.397%). However, we found a trend towards lower incidence (0.113%) in our whole cohort (RR 0.28, 95%CI 0.04–2.01 for the whole cohort compared to UK population). The trend towards lower incidence in this cohort could be because of prior education regarding general infection risk and response to public health messages. While risk stratification and shielding could be effective, prior education regarding general infection risk and public health messages to enhance health protection behaviours during a pandemic may have equal or more important roles.

**Table:** 

**Key Points** • *Patients on treatment for rheumatic disorders showed a trend for lower incidence of COVID-19 transmission irrespective of shielding letter status* • *This could potentially be because of prior education regarding infection risk received when starting on disease-modifying medication* • *Health education influencing health protection behaviours may be of equal or more importance than shielding information in reducing transmission of SARS-CoV-2*

**Key Points**

• *Patients on treatment for rheumatic disorders showed a trend for lower incidence of COVID-19 transmission irrespective of shielding letter status*

• *This could potentially be because of prior education regarding infection risk received when starting on disease-modifying medication*

• *Health education influencing health protection behaviours may be of equal or more importance than shielding information in reducing transmission of SARS-CoV-2*

## Background

As the coronavirus 19 disease (COVID-19) pandemic, caused by the severe acute respiratory syndrome coronavirus 2 (SARS-CoV-2), took hold in the United Kingdom (UK) in early 2020, the government and Public Health England rapidly issued guidance regarding “shielding” so that those deemed most vulnerable if infected by the virus could take measures to reduce their chances of infection [1]. This involves strict self-isolation for patients within their home and minimising contact with others with whom they share a household.

Within rheumatology teams, we care for patients with a range of autoimmune disease who are frequently on immunosuppressive treatments. Therefore, the British Society for Rheumatology (BSR) issued additional guidance for rheumatology teams to assist in the risk stratification of their patients to identify those required to shield [2]. The guidance allocates a risk score to various immunosuppressive treatments and also to the presence of co-morbidities. Patients scoring ≥ 3 are deemed high risk and advised to shield. Here, we describe our experience of using this guidance, with adaptation to suit our patient population.

## Methods

The BSR risk stratification guidance was adapted for local use by omitting cyclophosphamide in scoring criteria as we had no patients on this treatment in our service at assessment. A cohort of patients requiring risk stratification was compiled by the rheumatology pharmacy team by identifying patients currently on prescription and monitoring databases for disease-modifying anti-rheumatic drugs (DMARDs), biologics, janus kinase (JAK) inhibitors and corticosteroids prescribed by the department.

A risk stratification assessment was then performed for these patients by reviewing rheumatology clinic letters on the Letters Database, and where necessary, online General Practitioner (GP) records and recent laboratory investigations. Patients reviewed in clinic from March 24 were also opportunistically risk assessed. Patients scoring ≥ 3 were sent a letter advising them of their high-risk status and advising them to shield. This was copied to their GP. The scores, underlying rheumatology diagnoses and other risk factors were collated and processed in Microsoft Access and Excel 2010.

As we approached 2 months into the period of shielding, the COVID-19 status of the total cohort of patients was reviewed by accessing electronic hospital laboratory records which show the outcome of tests performed within the trust. The outcome of COVID-19 antigen testing reported up until 27 May 2020 was recorded. Relative risks were calculated to compare the whole assessed cohort to Bolton and UK populations.

## Results

### Risk stratification score

**Fig. 1:**
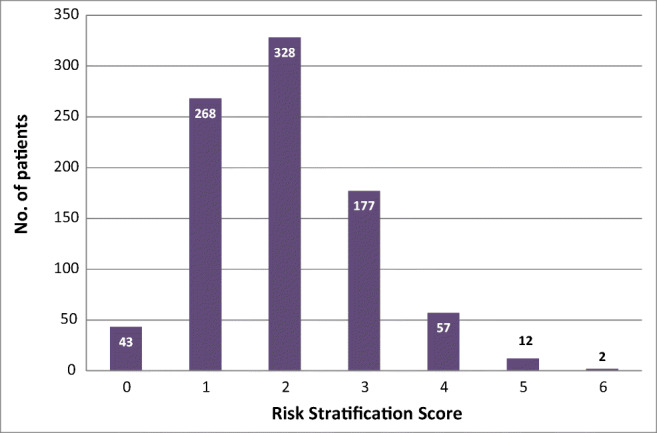
COVID-19 risk stratification score of the whole cohort assessed

The total number of patients assessed was 887. Out of these, 248 (28%) patients scored ≥ 3 and were sent a standard shielding letter. In this shielding letter group, the most common score was 3 with 177 (71.4%) patients (Fig. 1). The maximum score possible using our adapted risk stratification tool was 6 and there were two patients achieving this score.

### Risk factors

**Fig. 2:**
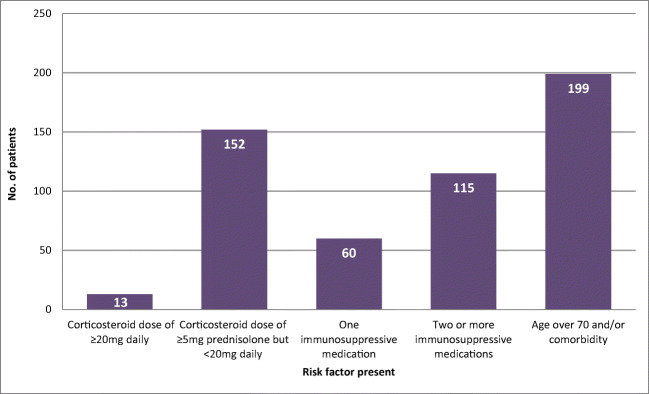
Risk factors in the shielding letter group

In the shielding letter group, the most common risk factor was age ≥ 70 years and presence of a listed co-morbidity (diabetes mellitus, pre-existing lung disease, renal impairment, history of ischaemic heart disease or hypertension), present in 199 (80.4%) of the patients (Fig. 2). The most common immunosuppressive medication taken by patients in the shielding letter group was lower dose steroids (prednisolone > 5 mg but < 20 mg) which were taken by 152 (61.3%) of the patients.

### Rheumatology diagnoses

**Fig. 3:**
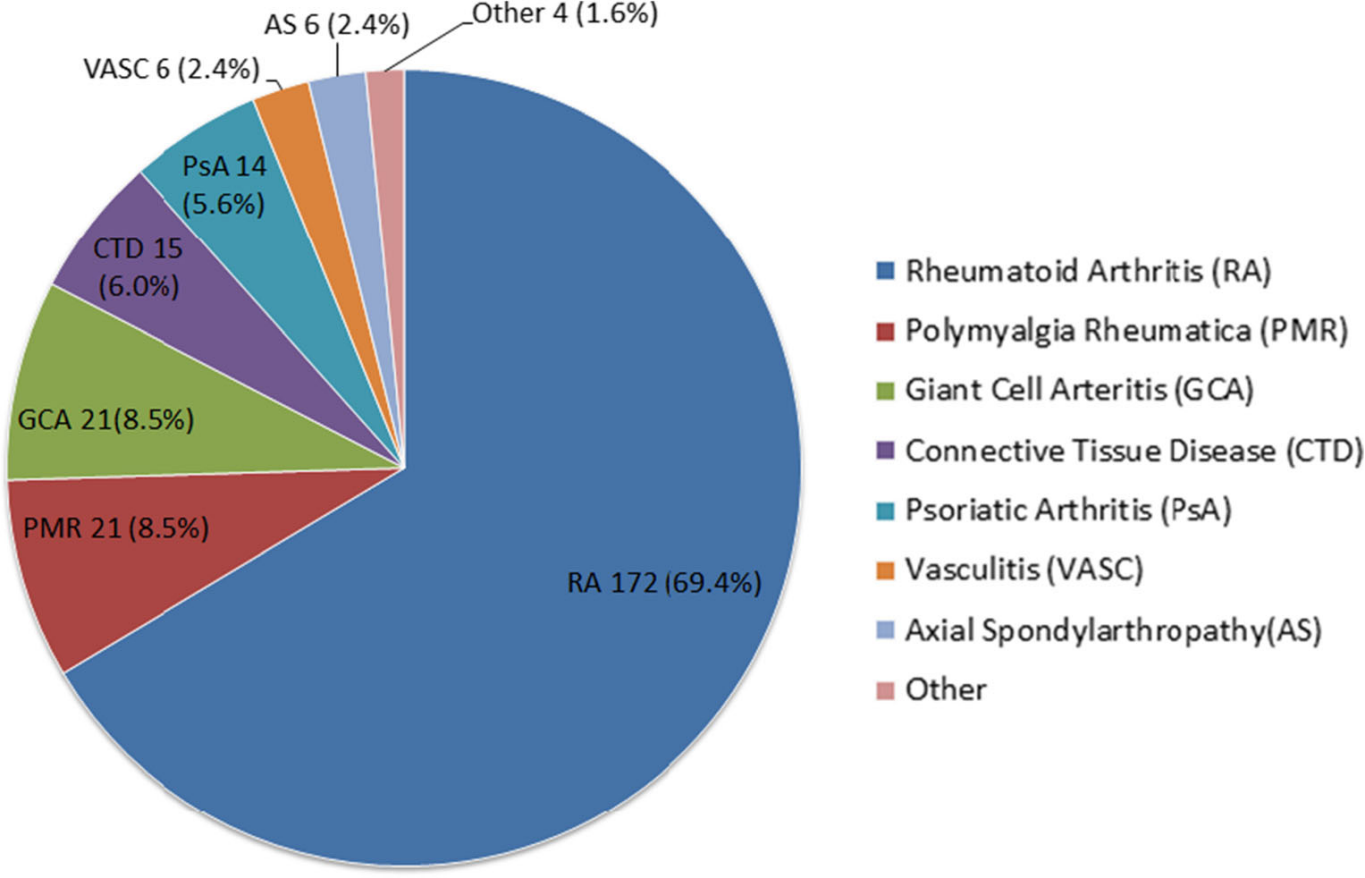
Distribution of rheumatology diagnoses in the shielding letter group

After the initial risk assessments were performed, records of patients in the shielding letter group were reviewed to identify their primary rheumatology diagnosis (Fig. 3). In 16 patients, there was more than one rheumatology diagnosis. The most common rheumatology condition was rheumatoid arthritis (69.4%). Polymyalgia rheumatica (8.5%) and giant cell arteritis (8.5%) were the next most prevalent. Connective tissue disease accounted for 6% of patients and this category included Sjogren’s syndrome, systemic lupus erythematosus, myositis, dermatomyositis and mixed connective tissue disorder in order of prevalence. There were 4 patients categorised as “Other” with diagnoses of reactive arthritis, sarcoidosis, IgG4-related disease and a patient taking steroids for Addison’s disease under the rheumatology service for osteoporosis. Of the two patients who had the maximum risk stratification score of 6, one had a diagnosis of rheumatoid arthritis and the other of myositis.

### COVID-19 status

Between 29 January and 27 May 2020, out of the 887 patients in the total cohort, 11 (1.24%) were identified as having swabs performed for SARS-CoV2, and 10 of these had negative results. The patient who tested positive was in the shielding letter group with a risk stratification score of 3. This gave coronavirus incidence rates of 0.403% and 0.113% in the shielding group and whole cohort assessed respectively. Data from Public Health England and the National Office of Statistics were used to calculate local and UK incidence rates for comparison (accessed on 27 May 2020, correct up to and including 26 May 2020) [3, 4]. Bolton had 1001 cases out of a population of 285,400 [5], giving an incidence rate of 0.351% (relative risk (RR) for whole cohort 0.32, 95%CI 0.05–2.28, *P* = not significant). The UK had 265,227 cases out of a population of 66,796,807 giving an incidence rate of 0.397% (RR for whole cohort 0.28, 95%CI 0.04–2.01, *P* = not significant).

## Discussion

This analysis included only those patients currently on the prescription and monitoring databases of the department. For patients on shared care with prescriptions generated in general practice, a letter was sent out with the risk stratification guidance, requesting GPs to carry out the assessments on patients identified on their databases.

A limitation in identification of patients tested was that any tests on out-of-area patients at other trusts were not visible on our system. As our service only has a small number of out-of-area patients and considering the static nature of Bolton’s population, this is unlikely to have had a significant effect on our results. We used a positive antigen test result as a surrogate marker for incidence, but as we used the same approach for both the whole assessed cohort, and the Bolton and UK populations, we expect this is unlikely to have a significant effect on the relative risks calculated. Antibody surveys in the future could potentially reveal different incidence levels.

There is growing opinion that stratified shielding should be recognised as a population health strategy [6]. However, when examining the SARS-CoV2 incidence rates, it is difficult to quantify the extent to which the shielding letter, and shielding itself, has effect on reducing transmission as we do not have a reference population of patients with the same risk profile who did not shield. Patients within our shielding letter group are thought to be at high risk of severe infection when compared to the general public. It could be expected that receiving the shielding letter would lead to effective shielding, and this in turn should result in a lower incidence in the shielding population.

It was reassuring to see that only one patient from the whole assessed cohort tested positive for SARS-CoV2. While it is difficult to draw firm conclusions from a single positive test in our study, the incidence rates in our shielding letter group and the general population were similar. However, in our whole screened cohort, there seems to be a trend towards lower incidence compared to the general population suggesting that patients, including those without a shielding letter, may have been practicing self-isolation and social distancing effectively. This may be because patients with chronic disease in general may be more attuned to public health messages through the media. However, it could be because of prior health education received by rheumatology patients regarding general infection risk at routine counselling when starting disease-modifying therapy. In line with this, Favalli et al. found that 90% of rheumatology patients in Milan reported that they had adopted health protection behaviours based on social distancing and use of personal protective equipment as preventative strategy against COVID-19 since the start of the pandemic [7]. There is also growing evidence and opinion that investment in health literacy of populations and organisations could flatten the curve for COVID-19 and other diseases [8].

Although prevalence of certain chronic conditions such as diabetes in patients with COVID-19 infection has been studied [9], there was initial paucity of published incidence of COVID-19 infection in patients with rheumatology conditions. A recent study in Hong Kong showed incidence of COVID-19 in rheumatology patients similar to incidence seen in their general population (0.0126% in patients with rheumatologic diseases, compared to 0.0142% in the general population) [10]. Their estimated incidences were much lower than those we are reporting in this study, but differences in local testing protocols and reporting methodology may mean that these results are not directly comparable. However, the fact that there was little difference between rheumatology patients and general population incidence in the Hong Kong study may imply that the adherence to health protection measures by Hong Kong’s general population was similar to those of their rheumatology patients, for example, earlier and very widespread adoption of face coverings [11]. This greater adherence to health protection behaviours at the general population level could potentially be a reflection of the health education response to the previous SARS 2002–2003 epidemic in the region. Further studies into COVID-19 incidence in patients, while taking into account health protection behaviours, may be useful to confirm and clarify the themes further. It would also be interesting to see future studies assess how incidence in high-risk patients changes when shielding measures cease and patients begin to re-integrate with the wider community. This could inform future strategies in preparing in the hiatus for further waves of this pandemic, and preventing others. At present, with focus divided on multiple and diverse strategies being explored by various national governments and health agencies in controlling COVID-19, there is a danger that the simple message of health education to enhance health protection behaviours of populations could be overlooked, as this could be an important strategy to minimise transmission.

## Conclusion

The incidence of COVID-19 in our assessed patient cohort showed a trend towards lower incidence than the general population rate irrespective of whether they were sent shielding letters. This could be because of greater prior awareness regarding general infection risk in this cohort, and response to public health messages. We therefore believe that, while risk stratification and shielding could be an effective method of safeguarding vulnerable chronic disease patients, prior education regarding general infection risk and public health messages to enhance health protection behaviours during a pandemic may have equal or more important roles. Further studies evaluating these themes could be valuable in informing future national policy and also wider public health strategy towards preventing and managing pandemics.
